# Signet Ring Cell Carcinoma of the Vater's Ampulla: A Very Rare Malignancy

**DOI:** 10.1155/2012/402798

**Published:** 2012-09-26

**Authors:** K. Daoudi, K. El Haoudi, N. Bouyahia, A. Benlemlih, S. Arifi, N. Mellas, K. Mazaz, A. Amarti, O. El Mesbahi

**Affiliations:** ^1^Medical Oncology Department, HASSAN II University Hospital, BP 7206, Fes Zohor, 30014 Fez, Morocco; ^2^Visceral Surgery Department, HASSAN II University Hospital, BP 7206, Fes Zohor, 30014 Fez, Morocco; ^3^Department of Pathology, HASSAN II University Hospital, BP 7206, Fes Zohor, 30014 Fez, Morocco

## Abstract

Most tumors affecting Vater's ampulla are adenocarcinomas, and other histological variants are less frequent. Signet ring cell carcinoma is more commonly found in the stomach than at other sites of the digestive system. Signet ring cell carcinoma of the Vater's ampulla is extremely rare, and only 15 cases have previously been described in the literature. It mainly occurs in elderly patients (median age = 57 years). We report a case of advanced signet ring cell carcinoma of the ampulla of Vater, with invasion of the duodenum (D3) admitted in the Medical Oncology Unit of HASSAN II University Hospital.

## 1. Introduction 

Tumors of the ampulla of Vater are not common [1]. Ampulla of Vater cancers are usually well differentiated adenocarcinomas [2].

Signet-ring cell carcinoma (SRCC) usually occurs in the gastrointestinal tract. The World Health Organization (WHO) defines it as a special type or a variant of gastrointestinal adenocarcinoma. SRCs may exist alone or coexist with any other types of malignant gastrointestinal tumors. SRCC is very rarely found among carcinomas of the ampulla of Vater [3].

Here, we present new case with advanced SRCC in the ampulla of Vater, and we analyse different data about diagnosis, possible etiology, and treatment of this entity.

## 2. Case Presentation

We report the case of a 55-year-old man, without pathological antecedents, admitted with jaundice and abdominal pain appearing 6 months ago.

Abdominal ultrasound showed dilatation of the common bile duct and the intrahepatic bile ducts.

Abdominal computed tomography and Bili-IRM showed a tumor in the ampulla of Vater with invasion of D3 and a dilatation of the extra hepatic bile duct; no loco regional lymph nodes were observed.

Duodenoscopy showed irregularly shaped erosion on the ampulla of Vater and histology of the biopsy revealed a poorly differentiated signet ring cell carcinoma.

A cephalic duodenopancreatectomy and extended lymphadenectomy were performed.

The definitive histological study showed a signet ring cell carcinoma of the Vater's ampulla with negative resection margins.

Postoperative thoraco-abdomino-pelvic computed tomography, CA19-9, and CEA were normal.

The tumor was classified as pT3 N0 M0.

6 cycles of adjuvant chemotherapy based on CISPLATIN + GEMCITABINE were performed with good clinical tolerance and nonfebrile neutropenia grade 3 after the second cycle.

The patient remained well and had no evidence of loco regional and metastatic recurrence during the 8 months of followup.

## 3. Discussion 

SRCC can arise in many organs, but it usually occurs in the gastrointestinal tract, especially in the stomach. It has been reported that 90% of SRCC occurs in the stomach, with the rest arising in several other organs, including the breast, gallbladder, pancreas, urinary bladder, and colon [4].

It is extremely uncommon in the ampulla of Vater. Less than 15 cases have been described in the literature.

The presence of this kind of tumor has no clear histological explanation. 

Two possible theories have been proposed: the presence of gastric heterotopia in the ampulla of Vater or the existence of a perivaterian duodenal heterotopia of ulcerous etiology as the origin of a signet ring cell tumor which secondarily invades the ampulla of Vater [4].

Akatsu et al. [5] have summarized the previous 14 cases (eight men and six women) and concluded that the median age at diagnosis was 57 years (range, 32–83 years), approximately 15 years older than SRCC of the stomach, but similar to the median age for SRCC of the large bowel.

Hara et al. [6] report the case of seven patients presented SRCC of the Vater's ampulla. The mean age of the published cases was 60.3 years (range: 25–72 years). Jaundice was the most common symptom (5 out of 7 patients); the macroscopic appearance was superficial protruding (4 cases), ulcerative (2 cases), or diffuse infiltrative (1 case). The treatment was duodenopancreatectomy in six patients and local excision in one high-risk surgical patient. None of the resected patients had lymph node involvement.

 According to the TNM classification, they were divided between T3N0M0 (5 cases), T2N0M0 (1 case), and TxNxM0 (1 case).

The present patient had the typical characteristics of other cases in terms of age, jaundice, macroscopic appearance, treatment approach, and TNM classification.

For diagnosis of SRCC, helical computed tomography (CT) shows a dilated CBD (common bile duct) without a mass lesion in the ampulla of Vater in some cases [7–9].

In our case, abdominal computed tomography showed a tumor in the ampulla of Vater with invasion of D3 and a dilatation of the extra hepatic bile duct.

Contrast-enhanced ultrasound (CEUS) has gained increasing interest in recent years. By CEUS, the lesion may be displayed much clearer than by conventional gray-scale ultrasound. It can also offer a good method in the discrimination of ampullary carcinoma from nonmalignant lesions [10]. 

As for prognosis, signet ring cell tumors localized in other digestive organs have a poor prognosis. The scant number of cases reported in the ampulla precludes any conclusions about survival associated with this histological variant.

But lymph node involvement is a determinant prognostic factor in cancer of the ampulla of Vater [6]. 

Duodenopancreatectomy with pylorus preservation is the treatment of choice in ampullar cancers [6]. Adjuvant therapy has not showed survival benefit in patients without lymph node infiltration [2].

## 4. Conclusion

In conclusion, we have presented a rare case of signet ring cell carcinoma in the ampulla of Vater with invasion of the duodenum. Although several cases have been reported, the detailed clinicopathological features and prognosis are not clear. Additional reports are warranted.
